# Integration of a bacterial gene sequence into a chronic eosinophilic leukemia patient’s genome as part of a fusion gene linker

**DOI:** 10.1186/s40364-017-0101-z

**Published:** 2017-06-05

**Authors:** Saveen Sidhoo, Jesusa L. Rosales, Ki-Young Lee

**Affiliations:** 1 Arnie Charbonneau Cancer Institute, Departments of Cell Biology & Anatomy, University of Calgary, Calgary, AB T2N 4N1 Canada; 2 Biochemistry & Molecular Biology, University of Calgary, Calgary, AB Canada

**Keywords:** Leukemia, Cdk5rap2, PDGFRα

## Abstract

Analysis of databases from the human genome project (HGP), the 1000 Genomes Project (1KGP), and The Cancer Genome Atlas (TCGA) revealed bacterial DNA integration into the human somatic genome, particularly in tumor tissues. Fusion genes have also been associated with tumorigenesis and 34 PDGFR fusion genes are linked to hematological malignancies. Here, we determined that a 17-bp homologous sequence in *Marinobacter sp.* Hb8, *Rhodococcus fascians* D188, *Rhodococcus sp.* PBTS2, *Micrococcus luteus* strain trpE16 and *M. luteus* NCTC 2665 integrates into the genome of a chronic eosinophilic leukemia patient as part of the linker for the novel *CDK5RAP2-PDGFRα* fusion gene. The resulting fusion protein that has CDK5RAP2’s self-activating domain and PDGFRa’s tyrosine kinase domain but lacks PDGFRa’s membrane-binding and ligand-dependent activation properties may act together with the integrated bacterial sequence to readily phosphorylate downstream targets, amplify proliferation signals and promote leukemic cancer progression.

Analysis of the human genome project (HGP), the 1000 Genomes Project (1KGP), and The Cancer Genome Atlas (TCGA) databases revealed integration of bacterial DNA into the human somatic genome and this is observed more frequently in tumor tissues than in normal tissues [1]. This is quite interesting as certain viruses such as hepatitis B virus, human immunodeficiency virus and human T-cell lymphotrophic virus type 1 induce carcinogenesis via integration into the host cell genome but there is no concrete data that demonstrates cancer development due to somatic mutation from bacterial gene insertion into the human genome. Nonetheless, integration of specific bacterial DNAs has been linked to specific types of cancer. For example, many paired reads among various stomach adenocarcinoma samples show definite *Pseudomonas-*like DNA integration into or near the 5′-UTR and 3′-UTR of a few transcriptionally upregulated proto-oncogenes. It was hypothesized that such integration causes mutations in the repressor binding site, resulting in elevated expression and carcinogenesis. Moreover, thousands of read pairs were found to display indiscriminate *Acinetobacter-*like DNA integration into the mitochondrial genome of tissue samples from acute myeloid leukemia patients, further supporting the notion that integration of bacterial DNA into the human somatic genome may be involved in the development and/or progression of cancer.

A patient with chronic eosinophilic leukemia was found carrying a novel mRNA with an ins(9;4)(q33;q12q25) encoding for CDK5RAP2-PDGFRα fusion protein [2] (Fig. 1a). The *CDK5RAP2-PDGFR*α fusion event involves insertion of a 40-bp junction sequence between the *CDK5RAP2* and *PDGFRα* breakpoints. The junction consists of a 22-bp inverted intron 9 of *PDGFR*α and an 18-bp region from an unknown source. By NCBI BLAST Search, we found that the 18-bp is 100% identical to a sequence in *Marinobacter sp.* Hb8 (Fig. 1b). In addition, of the 18-bp component, 17-bp (2–18) is 100% identical to a sequence in *Rhodococcus fascians* D188, *Rhodococcus sp.* PBTS2, *Micrococcus luteus* strain trpE16 and *Micrococcus luteus* NCTC 2665, (Fig. 1b). Indeed, while host leukocytes phagocytose and release toxins to destroy bacteria, the latter’s genetic material may escape destruction and insert into the host genome via a transposon. The *AluSz6* retrotransposon in *PDGFR*α intron 10 (Fig. 1c) may facilitate insertion of the 18-bp *Marinobacter sp.* Hb8 sequence or the 17-bp *R. fascians* D188, *Rhodococcus sp*. PBTS2, *M. luteus* strain trpE16 or the *M. luteus* NCTC 2665 sequence into the fusion gene via misaligned recombination. The 18th bp (gray) may likely be added during fusion.

**Fig. 1:**
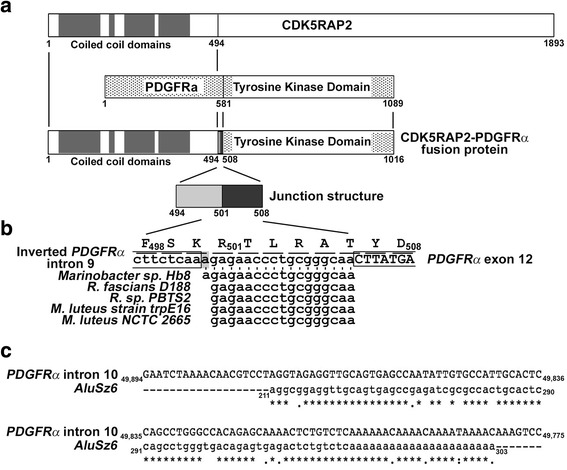
Insertion of the 18-bp sequence into the *CDK5RAP2*-*PDGFRα* fusion gene. **a**. Schematic diagram showing the structures of full-length CDK5RAP2, full-length-PDGFRα, and CDK5RAP2-PDGFRα fusion protein with its junction structure between CDK5RAP2 and PDGFRα. The N-terminal (aa 1 to 494) of CDK5RAP2 (exons 1–13), containing the coiled-coil domains (*dark gray boxes*), fuses with the C-terminal (aa 581 to 1089) of PDGFRα (exons 12–22), containing the tyrosine kinase domain, resulting in the 1016 aa CDK5RAP2-PDGFRα fusion protein. The 40-bp junction is composed of a 22-bp *PDGFR*α inverted intron 9 (aa 494–501, *light gray box*) and an 18-bp from an unknown source (aa 501–508). **b**. NCBI BLAST Search revealed that the18-bp is 100% identical to a sequence in *Marinobacter sp.* Hb8 complete genome (CP017715.1). Of the 18-bp, 17-bp (2–18) is 100% identical to a sequence in *Rhodococcus fascians* D188 complete genome (CP015235.1), *Rhodococcus sp.* PBTS2 complete genome (CP015220.1), *Micrococcus luteus* strain trpE16 genome (CP007437.1) and *Micrococcus luteus* NCTC 2665 complete genome (CP001628.1). The “a” (*gray*) is likely added during the fusion event, which together with “aa” in the inverted intron 9 sequence encodes K that does not exist in PDGFRα. **c**. Sequence alignment of intron 10 from *PDGFRα* [GenBank Accesssion # NP_006206.4] with *AluSz6* retrotransposon [Accession # DF0000052]. AluSz6 was found using Dfam 2.0 database. Sequences were aligned by CLUSTAL OMEGA 1.2.2 Multiple Sequence Alignment software. Nucleotide numbers are indicated on either side. Asterisks denote identical sequences between *hPDGFRα* and *AluSz6*. Broken lines represent missing nucleotide sequences in *AluSz6*

Fusion protein generation involves fork stalling and template switching. The multiple palindromic sequences in *PDGFRα* intron 9 could stall a replication fork, and homologous regions between *PDGFRα* intron 9 and *CDK5RAP2* intron 13 could facilitate template switching. In the *CDK5RAP2-PDGFRα* fusion gene, the truncated *PDGFRα* exon 12 containing the entire tyrosine kinase domain lacks aa1-aa581, which is replaced by *CDK5RAP2*’s N-terminal coiled coil domain (Fig. 1a) that could self-dimerize. Thus, the resulting CDK5RAP2-PDGFRα fusion protein lacks both membrane-binding ability and ligand-dependent activation. This cytosolic self-activated fusion protein kinase may, therefore, readily phosphorylate downstream targets, amplify proliferation signals and cause uncontrolled cell growth. Our premise is that the translated 18-bp region, RTLRA, wherein the 17-bp sequence encodes for in-frame amino acid sequence that is identical to *R. fascians* histidine kinase, regulates CDK5RAP2-PDGFRα function to induce leukemic cell growth. In fact, fusion genes contribute to all malignancies [3] and 34 PDGFR fusion genes have now been linked to hematological malignancies [4]. Potentially, other PDGFR fusion genes with the 18-bp bacterial gene segment could arise.

Indeed, lateral gene transfer from *Marinobacter sp.* Hb8, *R. fascians* D188, *Rhodococcus sp*. PBTS2, *M. luteus* strain trpE16 or the *M. luteus* NCTC 2665 into the somatic human genome is not surprising. Further analysis of the study by Riley et al. [1] revealed read pairs from TCGA that indicate the presence of Marinobacter, Rhodobacteraceae and Micrococcaceae sequences in genome samples of patients with acute myeloid leukemia. Micrococcaceae sequence was also detected in patients with lung squamous cell carcinoma, lung adenocarcinoma, breast invasive carcinoma, kidney renal clear cell carcinoma and kidney renal papillary cell carcinoma. However, in this letter, we report the first bacterial sequence integration into the genome of a patient with chronic eosinophilic leukemia. While the actual origin of the bacterial sequence is unknown, and there is yet no conclusive evidence that bacterial gene integration into the human somatic genome initiates carcinogenesis, it is likely that such integration alters the encoded CDK5RAP2-PDGFRα protein function to promote leukemic cell growth. This premise is consistent with the implied role of PDGFR fusion proteins in hematological cancer progression.

Since it is possible that identified bacterial gene integrations into the human genome can result from laboratory-based artifacts such as from chimeric DNA that could arise during library construction, it is critical to discriminate artifacts from true bacterial insertions. Riley et al. [1] have indicated different approaches on how to distinguish real bacterial integrations from laboratory artifacts. In the current study, the novel CDK5RAP2-PDGFRA fusion gene [2] that we examined was discovered by fluorescent in-situ hybridization (FISH), rapid amplification of cDNA ends-polymerase chain reaction (RACE-PCR) and sequencing. Since library construction was not involved in the discovery of the fusion gene that contains the bacterial gene sequence, it is unlikely that the identified bacterial integration is a laboratory-based artifact.

Thus, we have identified the 17-bp component of the linker for the *CDK5RAP2-PDGFRα* fusion gene in a chronic eosinophilic leukemia patient as a homologous sequence in *Marinobacter sp.* Hb8, *R. fascians* D188, *Rhodococcus sp*. PBTS2, *M. luteus* strain trpE16 and the *M. luteus* NCTC 2665. To our knowledge this is the first report on bacterial sequence integration into a chronic eosinophilic leukemia patient’s genome. We also mark the first account on integration of a bacterial sequence specifically into a fusion gene, which in this case is *PDGFRα*, an implied oncogenic driver in a number of leukemia patients.

## Abbreviations

CDK5RAP2: Cyclin-dependent kinase 5 (CDK5) regulatory subunit associated protein 2
PDGFRα: Platelet-derived growth factor receptor alpha
TCGA: The Cancer Genome Atlas
